# All-trans retinoic acid 45 mg/m^2^ is superior to 25 mg/m^2^ as the first induction regimen for the treatment of acute promyelocytic leukaemia: a retrospective analysis in a real-world clinical setting

**DOI:** 10.1038/s41408-021-00411-9

**Published:** 2021-01-29

**Authors:** Xinhui Zhang, Shanglong Feng, Jin Xu, Na Zhao, Xing Hu, Li Zhou, Juan Tong, Lei Xue, Lei Zhang, Yongsheng Han, Xingbing Wang, Liangquan Geng, Xiaoyu Zhu, Baolin Tang, Huilan Liu, Weibo Zhu, Xiaoyan Cai, Xin Liu, Zimin Sun, Changcheng Zheng

**Affiliations:** 1 Department of Hematology, Anhui Provincial Hospital, Anhui Medical University, Hefei, China; 2 Department of Hematology, the First Affiliated Hospital of USTC, Division of Life Sciences and Medicine, University of Science and Technology of China, Hefei, China; 3 Wannan Medical College, Wuhu, China

**Keywords:** Acute myeloid leukaemia, Translational research

Dear Editor,

Acute promyelocytic leukaemia (APL) is a distinctive type of acute myeloid leukaemia (AML), accounting for approximately 10% of AML^1^. All-trans retinoic acid (ATRA) has been used to treat APL in large well-designed clinical trials, which showed that the CR rate and long-term survival increased to 90% based on ATRA combined with arsenic, and the risk of early mortality during induction was only 2.5%~5%^2–4^. However, in the real-world clinical setting, early mortality is still high, with a range of 17–21.8%^4–8^. At present, there are two main doses of ATRA used in the world. In most haematology-oncology centres in Europe and the United States, the dosage of ATRA is 45 mg/m^2^/day, while the recommended dosage in China is 25 mg/m^2^/day^9–11^; both of them have good clinical effects. However, there is no relevant study demonstrating the differences between the two dosages of ATRA. To further clarify whether there is any difference in the clinical efficacy of the two dosages of ATRA for APL, we retrospectively analysed the data of APL patients in our centre in the real-world clinical setting and compared the efficacy between patients administered a dosage of ATRA of 25 mg/m^2^/day (ATRA-25 mg) and those administered a dosage of ATRA of 45 mg/m^2^/day (ATRA-45 mg), with a focus on the occurrence of differentiation syndrome, early death, disease relapse and long-term survival, to provide references for further improving the clinical efficacy of APL.

This study protocol was approved by the ethics committee of Anhui Provincial Hospital and was conducted in accordance with the Declaration of Helsinki. All patients received oral ATRA (a daily divided oral dose of 25 mg/m^2^ or 45 mg/m^2^) with or without arsenic trioxide (ATO) (a daily intravenous dose of 0.16 mg/kg) until CR. From January 2010 to December 2018, a total of 161 patients with newly diagnosed APL presented to our hospital; 132 patients were treated with an ATRA 25 mg/m^2^/day-based regimen (ATRA-25 mg group), while 29 patients were treated with an ATRA 45 mg/m^2^/day-based regimen (ATRA-45 mg group). Ninety patients also received ATO (ATRA plus ATO), including 62 patients in the ATRA-25 mg group and 28 patients in the ATRA-45 mg group (Supplementary Table 1S). Chemotherapy with idarubicin 8–12 mg/m^2^/day or daunorubicin 45–60 mg/m^2^/day for 3–4 days was added for high-risk patients, and for low-risk or intermediate-risk patients when necessary (especially for patients with WBC increasing more than 5 × 10^9^/L). Once diagnosed with differentiation syndrome^12–14^, dexamethasone at a dose of 10 mg twice daily was immediately initiated until symptoms improved.

The main clinical characteristics of these patients at the first diagnosis are summarised in Table 1. Based on the Sanz risk score^15^, 41 patients (31.1%) had high-risk disease and 91 patients (68.9%) had low- or intermediate-risk disease in the ATRA-25 mg group, while 11 patients (37.9%) had high-risk disease and 18 patients (62.1%) had low- or intermediate-risk disease in the ATRA-45 mg group.

**Table 1:** Clinical characteristics.

	ATRA-25 mg (*n* = 132)	ATRA-45 mg (*n* = 29)	*p*
Median age, years (range)	41 (9–74)	43 (18–71)	0.66
Age ≥ 14 years, No. (%)	127 (96.2)	29 (100)	
Sex			0.15
Male, No. (%)	72 (54.5)	11 (37.9)	
Female, No. (%)	60 (45.5)	18 (62.1)	
Median white blood cell, ×10^9^/L (range)	2.94 (0.29–220.86)	4.41 (0.34–135.87)	0.35
Median platelet, ×10^9^/L (range)	20 (2–179)	20 (3–60)	0.61
Fibrinogen level, g/L (range)	1.42 (0.35–4.77)	1.62 (0.34–3.32)	0.61
Sanz risk			0.78
Low-risk, No. (%)	19 (14.4)	3 (10.3)	
Intermediate-risk, No. (%)	72 (54.5)	15 (51.7)	
High-risk, No. (%)	41 (31.1)	11 (37.9)	
PML-RARA/ABL, % (range)	19.92 (1.60–112.68)	22.20 (2.18–39.88)	0.99
Cytogenetics	100 (75.6)	23 (79.3)	0.42
t(15;17), No. (%)	52 (39.4)	16 (55.2)	
t(15;17) plus other cytogenetic abnormalities, No. (%)	16 (12.1)	1 (3.4)	
Normal, No. (%)	26 (19.7)	5 (17.2)	
Others, No. (%)	6 (4.5)	1 (3.4)	
Median time to sustained platelet count ≥ 30 × 10^9^/L, days (range)	10 (1–32)	12 (3–20)	0.11
Median time to the peak WBC, days (range)	7 (2–19)	6 (3–14)	0.43
Differentiation syndrome, No. (%)	38 (28.8)	6 (20.7)	0.49
Follow-up among survivors in months, median (range)	60 (19–123)	38 (20–120)	0.098

*PML-RARA/ABL* Promyelocytic leukemia/retinoic acid receptor alpha/ABL

One hundred ten patients (68.3%) had platelet counts less than 30 × 10^9^/L at the first diagnosis and received a transfusion of platelets. The median platelet transfusion dose was 4 units (1–14) in the ATRA-25 mg group and 5 units (1–19) in the ATRA-45 mg group (*p* = 0.11) (Supplementary Fig. 1A). The median time to achieve a sustained platelet count greater than 30 × 10^9^/L was 10 days (1–32) in the ATRA-25 mg group and 12 days (3–20) in the ATRA-45 mg group (*p* = 0.11). The median fibrinogen level on admission was 1.42 g/L (0.35–4.77) in the ATRA-25 mg group and 1.62 g/L (0.34–3.32) in the ATRA-45 mg group (*p* = 0.61). The median fibrinogen concentrate infusion dose was 6.0 g (0.5–36) in the ATRA-25 mg group and 8.0 g (2.0–32) in the ATRA-45 mg group (*p* = 0.051) (Supplementary Fig. 1B). The median WBC count on admission was 2.94 × 10^9^/L (0.29–220.86) in the ATRA-25 mg group and 4.41 × 10^9^/L (0.34–135.87) in the ATRA-45 mg group (*p* = 0.35). For high-risk patients, the median time from diagnosis to the peak WBC count was 7 days (range: 2–19) in the ATRA-25 mg group and 6 days (range: 3–14) in the ATRA-45 mg group (*p* = 0.43). Differentiation syndrome occurred in 38 (28.8%) patients in the ATRA-25 mg group, including chest tightness or dyspnoea (*n* = 27), fever (*n* = 13), pleural effusion (*n* = 11), oedema (*n* = 7), renal failure (*n* = 3) and pulmonary oedema (*n* = 1). Six (20.7%) patients developed differentiation syndrome in the ATRA-45 mg group, including chest tightness or dyspnoea (*n* = 4), pleural effusion (*n* = 2), oedema (*n* = 1) and hypotension (*n* = 1). There was no significant difference between the two groups (*p* = 0.49).

After the first induction treatment, 108 patients (81.8%) in the ATRA-25 mg group and 28 patients (96.9%) in the ATRA-45 mg group achieved morphological complete remission (CR), which indicates that patients in the ATRA-45 mg group more easily achieved morphological CR (*p* = 0.049) (Supplementary Fig. 2A). After the second induction therapy, another 10 patients in the ATRA-25 mg group achieved morphological CR. Forty-nine patients (49/90, 54.4%) in the ATRA-25 mg group and 14 (14/23, 60.9%) patients in the ATRA-45 mg group achieved molecular CR after one course of induction treatment (*p* = 0.64) (Supplementary Fig. 2B).

There were 14 patients (10.6%) who died within the first 30 days of diagnosis in the ATRA-25 mg group. Intracranial haemorrhage (*n* = 10, 71.4%) was the most common cause of death, followed by differentiation syndrome (*n* = 2, 14.3%), severe infection (*n* = 1, 7.1%) and multiple organ failure (*n* = 1, 7.1%). However, in the ATRA-45 mg group, only 1 patient died due to differentiation syndrome on the 15th day of treatment. For all patients, the cumulative incidence of early death was 10.6% (95% Cl: 5.2–15.7%) in the ATRA-25 mg group and 3.4% (95% Cl: 0–9.9%) in the ATRA-45 mg group (*p* = 0.23) (Supplementary Fig. 3A). There were no differences in the early death rate between the two groups for the low- or intermediate-risk patients [7.7% (95% Cl: 2.1–13.0%) vs 5.6% (95% Cl: 0–15.6%), *p* = 0.74] (Supplementary Fig. 3B); however, for high-risk patients, there was a lower trend of early mortality in the ATRA-45 mg group (0%) than in the ATRA-25 mg group [17.1% (95% Cl: 4.7–27.8%)] (*p* = 0.15) (Supplementary Fig. 3C). For patients receiving ATRA plus ATO, the cumulative incidence of early death was 4.8% (95% Cl: 0–10.0%) in the ATRA-25 mg group and 3.6% (95% Cl: 0–10.2%) in the ATRA-45 mg group (*p* = 0.79) (Supplementary Fig. 3D).

Of the patients who achieved CR in the ATRA-25 mg group (*n* = 118), 17 patients experienced relapse during follow-up (including eight haematological relapses, five haematological and CNS relapses, and four isolated CNS relapses). Until now, none of the patients in the ATRA-45 mg group have relapsed. For the whole cohort, the 5-year cumulative incidence of relapse was lower in the ATRA-45 mg group (0%) than in the ATRA-25 mg group [16.6% (95% Cl: 9.7–24.9%)] (*p* = 0.046) (Supplementary Fig. 3E). For low- or intermediate-risk patients, the 5-year relapse rate was 0% in the ATRA-45 mg group and 12.9% (95% Cl: 6.2–22.1%) in the ATRA-25 mg group (*p* = 0.19) (Supplementary Fig. 3F). For high-risk patients, the 5-year relapse rate was 0% in the ATRA-45 mg group and 27.0% (95% Cl: 10.4–46.8%) in the ATRA-25 mg group (*p* = 0.099) (Supplementary Fig. 3G). For patients receiving ATRA plus ATO, the cumulative incidence of relapse was significantly lower in the ATRA-45 mg group (0%) than in the ATRA-25 mg group [26.5% (95% Cl: 5.7–42.8%)] (*p* = 0.024) (Supplementary Fig. 3H).

Among the patients still alive, the median follow-up time was 60 months (range, 19–123) for the ATRA-25 mg group and 38 months (range, 20–120) for the ATRA-45 mg group (*p* = 0.098). For the whole cohort, the overall survival (OS) in the ATRA-45 mg group was slightly higher than that in the ATRA-25 mg group: the 5-year OS rates of patients in the ATRA-45 mg group and the ATRA-25 mg group were 96.6% (95% Cl: 78.0–99.5%) and 84.0% (95% Cl: 76.1–89.4%), respectively (*p* = 0.095) (Fig. 1A). The event-free survival (EFS) in the ATRA-45 mg group was significantly higher than that in the ATRA-25 mg group: the 5-year EFS rates of patients in the ATRA-45 mg group and the ATRA-25 mg group were 96.6% (95% Cl: 78.0–99.5%) and 72.5% (95% Cl: 63.0–79.9%), respectively (*p* = 0.019) (Fig. 1B). For low- or intermediate-risk patients, OS and EFS were similar between the two groups. The 5-year OS was 94.4% (95% CI, 66.6–99.2%) for the ATRA-45 mg group and 87.9% (95% CI, 78.4–93.4%) for the ATRA-25 mg group (*p* = 0.53) (Fig. 1C); the 5-year EFS was 94.4% (95% CI, 66.6–99.2%) for the ATRA-45 mg group and 78.6% (95% CI, 67.5–86.3%) for the ATRA-25 mg group (*p* = 0.23) (Fig. 1D). For high-risk patients, the 5-year OS for patients in the ATRA-45 mg group was 100%, which was slightly higher than that in the ATRA-25 mg group [75.6% (95% CI, 59.4–86.1%)] (*p* = 0.081) (Fig. 1E), and the 5-year EFS for patients in the ATRA-45 mg group was 100%, which was significantly higher than that in the ATRA-25 mg group [58.1% (95% CI, 38.6–73.4%)] (*p* = 0.025) (Fig. 1F).

**Fig. 1:**
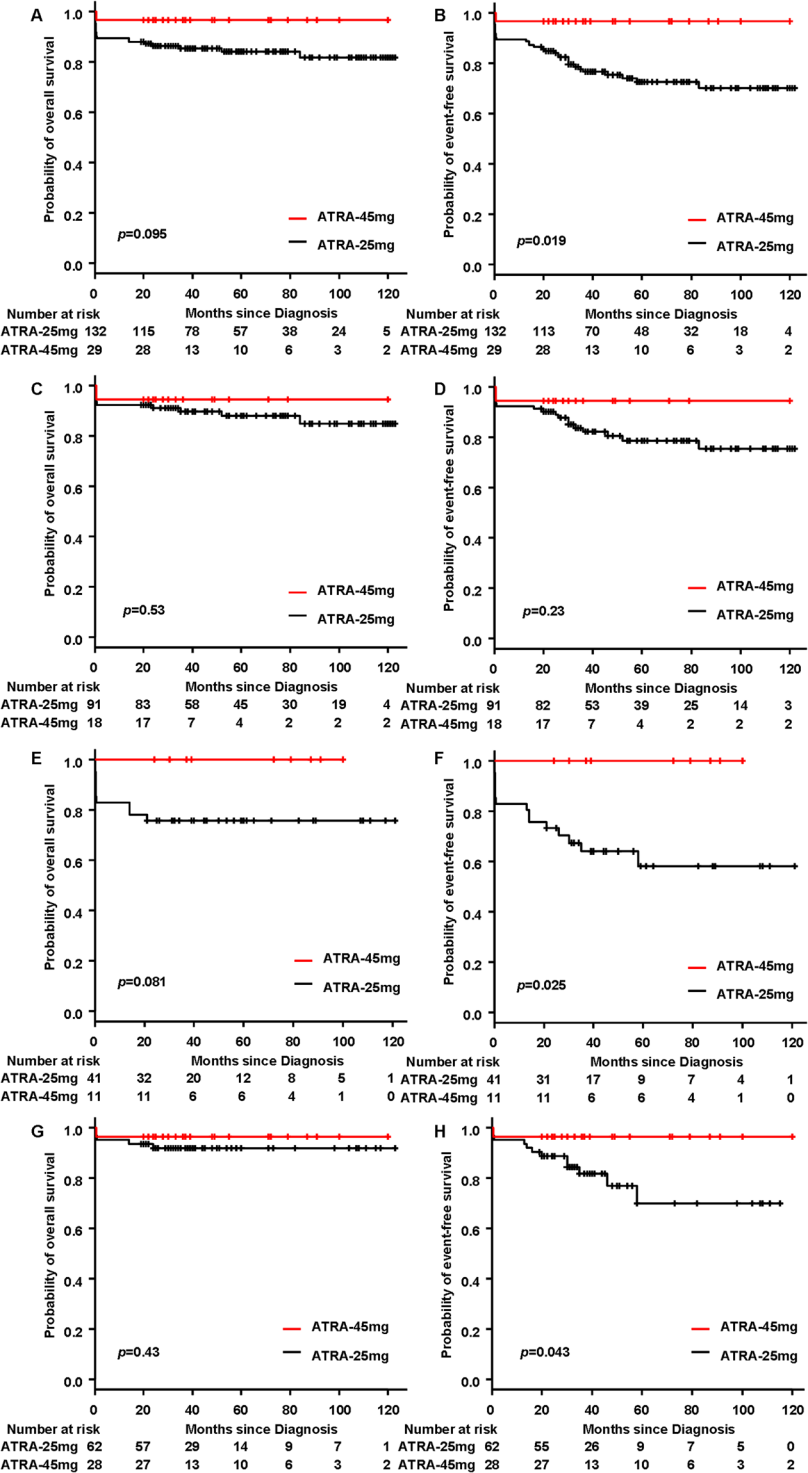
Overall survival and event-free survival. **A** Overall survival (OS) for the whole cohort. **B** Event-free survival (EFS) for the whole cohort. **C** OS for low- or intermediate-risk patients. **D** EFS for low- or intermediate-risk patients. **E** OS for high-risk patients. **F** EFS for high-risk patients. **G** OS for patients receiving ATRA plus ATO. **H** EFS for patients receiving ATRA plus ATO.

For patients receiving ATRA plus ATO, the 5-year OS was 96.4% (95% CI, 77.2–99.5%) in the ATRA-45 mg group, which was similar to that in the ATRA-25 mg group [91.7% (95% CI, 81.3–96.5%)] (*p* = 0.43) (Fig. 1G). However, the 5-year EFS for patients in the ATRA-45 mg group was 96.4% (95% CI, 77.2–99.5%), which was significantly higher than that in the ATRA-25 mg group [69.9% (95% CI, 48.1–84.0%)] (*p* = 0.043) (Fig. 1H). Early death, relapse and survival between patients receiving ATRA plus ATO- or ATRA-based induction is shown in Supplementary Fig. 4.

To our knowledge, this is the first clinical study to compare the clinical efficacy of ATRA-45 mg (45 mg/m^2^/day) vs ATRA-25 mg (25 mg/m^2^/day) for the treatment of APL in a real-world clinical setting. This study indicates that for APL, induction treatment based on ATRA 45 mg/m^2^/day could more easily lead to morphological CR and can achieve a higher long-term survival rate and a lower disease relapse rate than ATRA 25 mg/m^2^/day without increasing the risk of DS and coagulation dysfunction. However, there are inherent shortcomings in this study, including the small sample size of only 29 patients in the ATRA-45 mg group, the nonprospective and nonrandomized study design in a single centre, etc. On this basis, we plan to conduct a randomised controlled multicentre clinical trial to expand the sample size and further verify the clinical efficacy of ATRA 45 mg/m^2^/day for the treatment of contemporary APL.

## Supplementary information

Supplementary materials.
