# Supplementary material for: All-trans retinoic acid 45 mg/m2 is superior to 25 mg/m2 as the first induction regimen for the treatment of acute promyelocytic leukaemia: a retrospective analysis in a real-world clinical setting
Source: Blood Cancer J. 2021 Jan 29;11(1):15. doi: 10.1038/s41408-021-00411-9 (PMC7846739; doi:10.1038/s41408-021-00411-9)
Supplement: Supplementary file 1 — Supplementary materials. [file 41408_2021_411_MOESM1_ESM.doc]

|  | **ATRA+ATO based**  **(n=90)** | **ATRA based (n=71)** | ***p*** |
| --- | --- | --- | --- |
| **Median age, years(range)** | 41(9-74) | 43(12-72) | 0.62 |
| **Sex, No. (%)** |  |  | 0.99 |
| Male | 46 | 37 |  |
| Female | 44 | 34 |  |
| **Median white blood cell,×10^9^/L(range)** | 4.0(0.29-220.86) | 2.2(0.37-122.16) | 0.015 |
| **Median platelet,×10^9^/L(range)** | 20.5(4-123) | 18.0(2-179) | 0.48 |
| **Fibrinogen level (g/L)** | 1.50(0.34-4.02) | 1.44(0.35-4.77) | 0.61 |
| **Sanz risk** |  |  | 0.011 |
| Low-risk | 7 | 15 |  |
| Intermediate | 47 | 40 |  |
| High-risk | 36 | 16 |  |
| **Median time to sustained platelet**  **count ≥30×10^9^/L, days(range)** | 11(1-32) | 9(1-31) | 0.24 |
| **Median time to the peak WBC,** **days(range)** | 7(2-19) | 7.5(3-14) | 0.74 |
| **Differentiation syndrome, No. (%)** | 31 | 32 | 0.20 |
| **Morphological CR, No. (%)** | 81 | 52 | 0.007 |
| **Molecular CR, No. (%)** | 40 | 24 | 0.69 |
| **Follow-up among survivors, (months) , median (range)** | 39(14-123) | 78(14-122) | ＜0.001 |

**Supplementary Table 1S** Characteristic of patients with ATRA+ATO or ATRA based induction

**
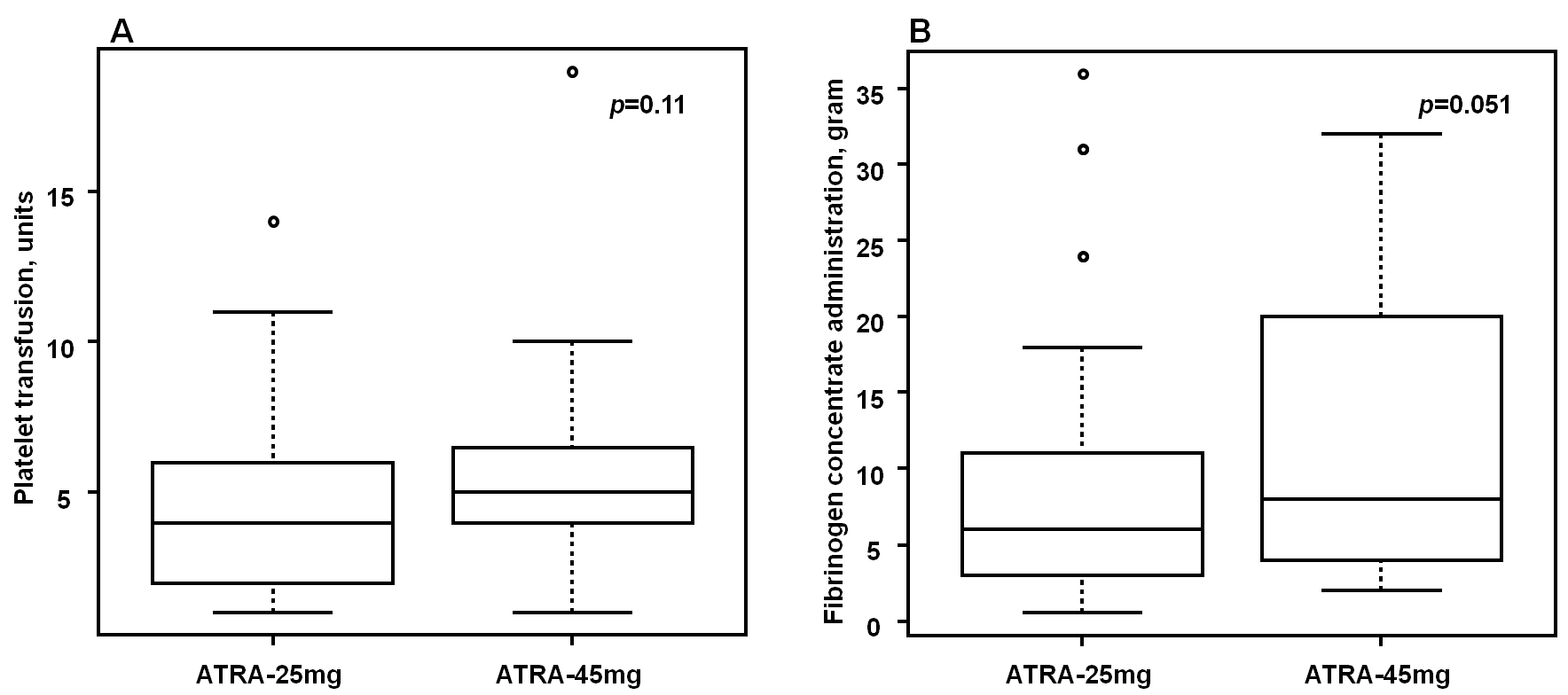
**

**Supplementary Figure 1 Platelet and fibrinogen transfusion**

A. Platelet transfusion. B. Fibrinogen transfusion

**
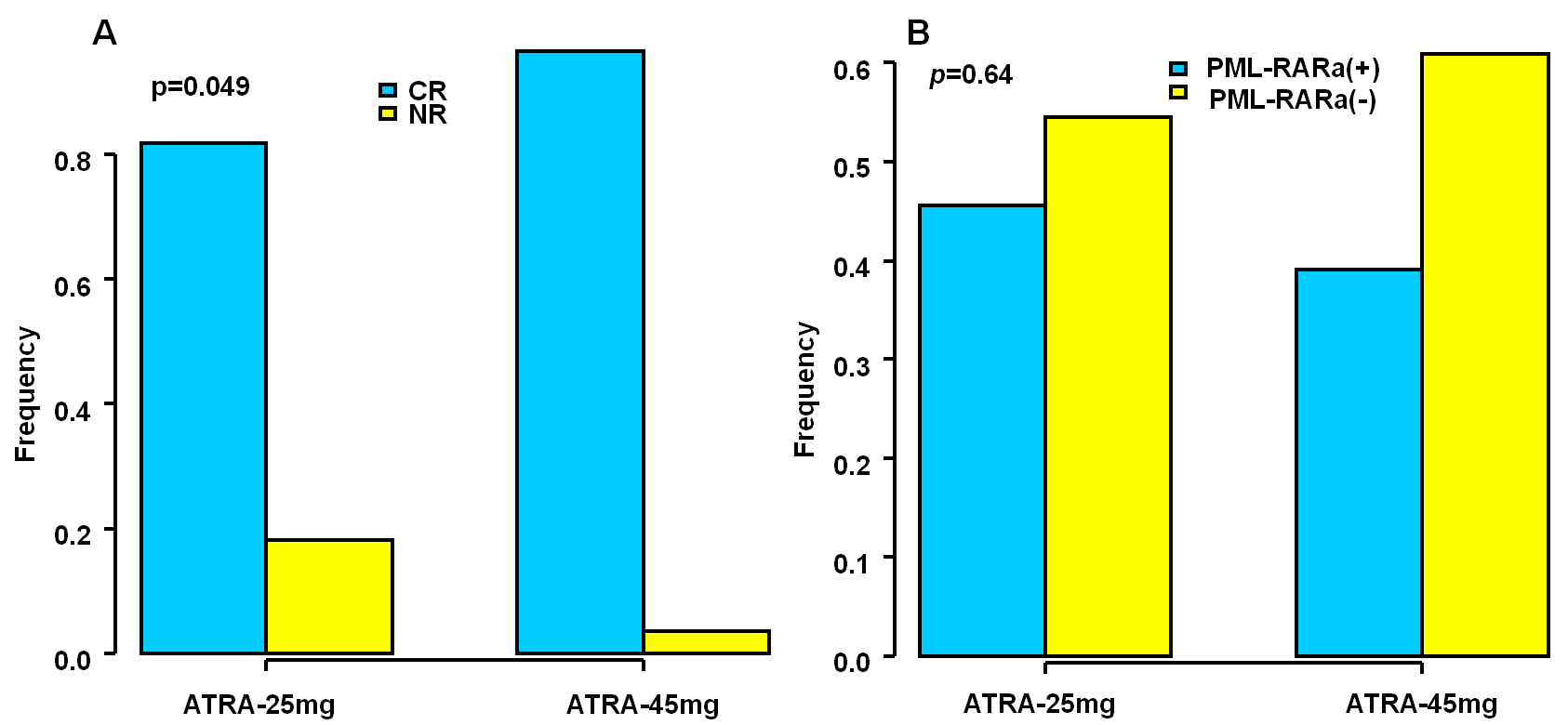
**

**Supplementary Figure 2 Complete remission after one course induction treatment**

a. Morphological complete remission (CR). b. Molecular CR

**
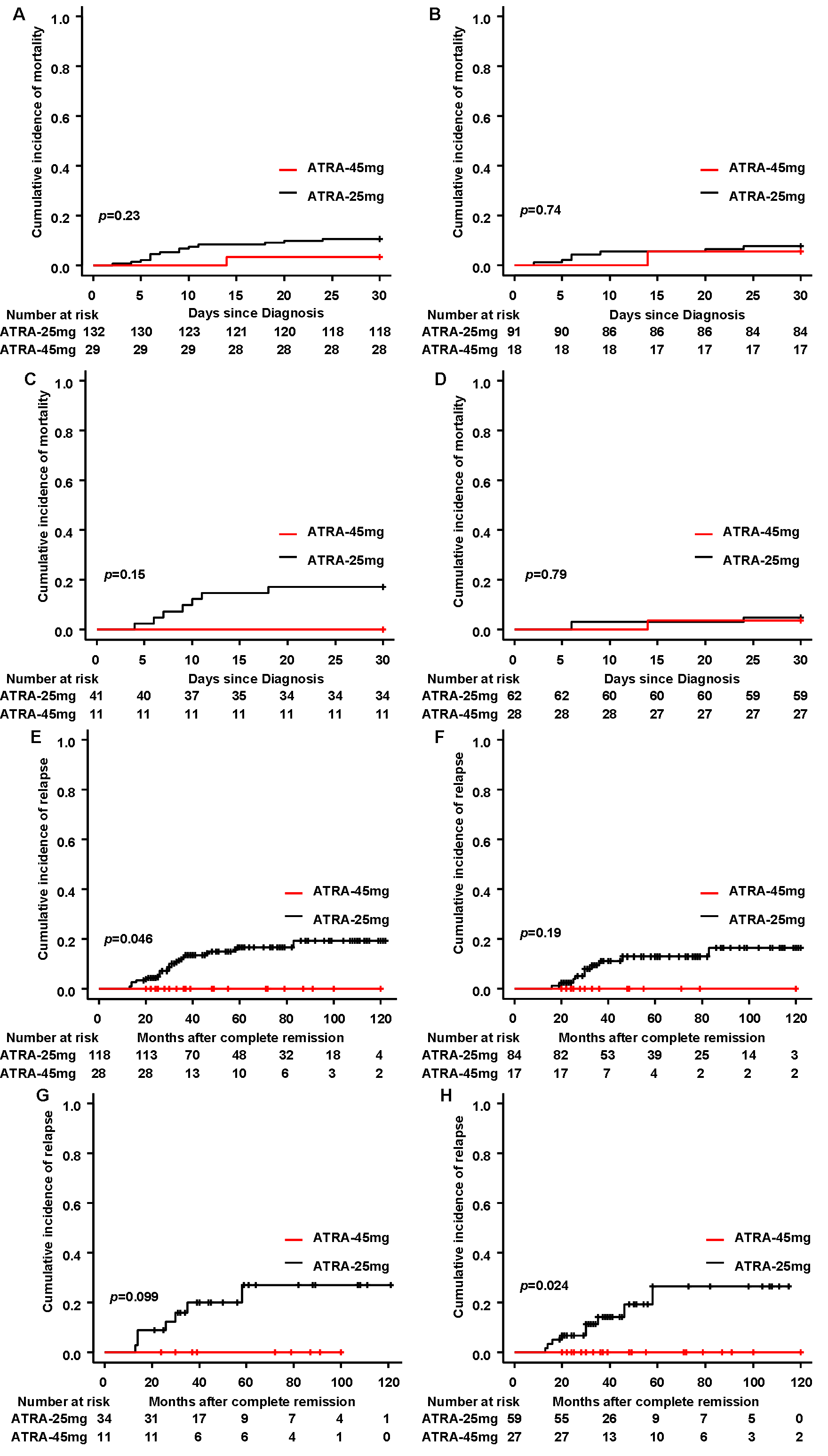
**

**Supplementary Figure 3 Early death and relapse**

a. Early death for the whole cohort. b. Early death for low or intermediate -risk patients. c. Early death for high-risk patients. d. Early death for patients receiving ATRA plus ATO. e. Relapse for the whole cohort. f. Relapse for low or intermediate -risk patients. g. Relapse for high-risk patients. h. Relapse for patients receiving ATRA plus ATO.

**
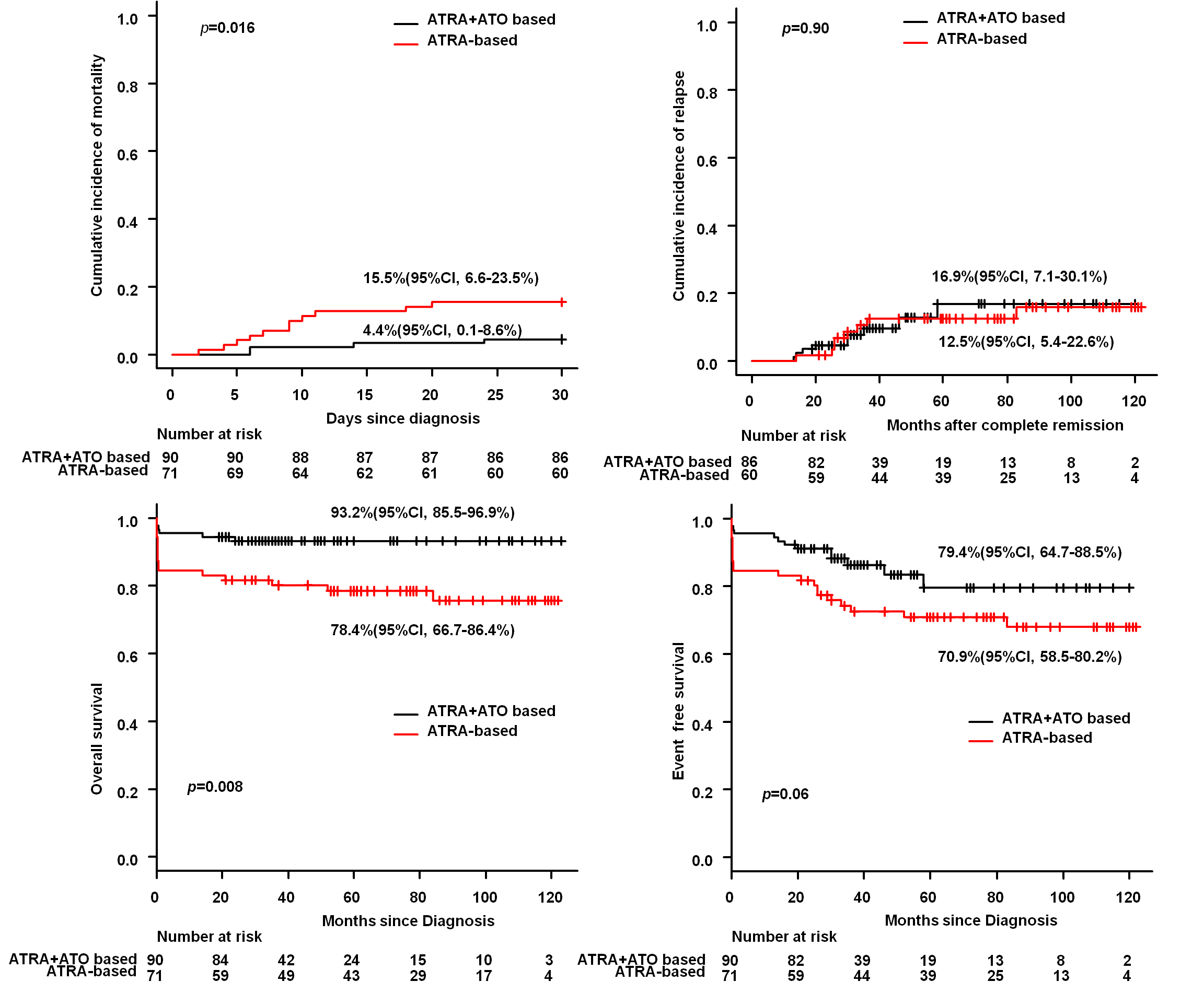
**

**Supplementary Figure 4 Early death, relapse and survival between patients treated with ATRA+ATO- or ATRA-based induction**

The cumulative incidence of early death in the first 30 days was 4.4% (95%Cl: 0.1-8.6%) in the ATRA+ATO-based induction group and 15.5% (95%Cl: 6.6%-23.5%) in the ATRA-based induction group (p=0.016); the 5-year relapse rate was 16.9% (95%Cl: 7.1%-30.1%) in the ATRA+ATO-based induction group and 12.5% (95%Cl: 5.4%-22.6%) in the ATRA-based induction group (p=0.90).

The OS in the ATRA+ATO-based induction group was significantly higher than the ATRA-based induction group: the 5-year OS for patients in the ATRA+ATO-based induction group and the ATRA-based induction group was 93.2% (95%Cl: 85.5%-96.9%) and 78.4% (95%Cl: 66.7%-86.4%), respectively (p=0.008). The EFS in the ATRA+ATO-based induction group was slightly higher than the ATRA-based induction group: the 5-year EFS for patients in the ATRA+ATO-based induction group and the ATRA-based induction group was79.4% (95%Cl: 64.7%-88.5%) and 70.9% (95%Cl: 58.5%-80.2%), respectively (p=0.06).
